# Voice for Vesna

**DOI:** 10.3325/cmj.2011.52.214

**Published:** 2011-04

**Authors:** Ana Marušić

*If it be now, 'tis not to come; If it be not to come, it will be now; If it be not now, yet it will come: the readiness is all.* William Shakespeare (1564-1616), Hamlet, Act V, scene ii

Vesna Andrijević Matovac left us on Valentine’s Day. She left us with the pain of love and loss and with wonder at how death was possible for her who was such a fighter, with not a day lost in the battle against cancer. As one of her friends said, Vesna died a valiant death, not from her disease but from the wounds inflicted in the battles she won.

The experiences of her struggle that she so generously shared with other patients were so vivid and encouraging. She did not leave the listener with a feeling of sorrow, dread, or embarrassment in the face of such grave disease as cancer and the certainty of death. One would feel like being comforted by a wise and trusted friend, guided over obstacles to a safe rest. Her story-telling power was the reason why I asked her to write for us. I knew her stories will find a way both to the patients and health care professionals.

For me personally, knowing and working with Vesna was a unique experience. We had many discussions about a number of academic issues, as she was a strong public voice in many activities related to Croatian science and technology development. But when we talked about her illness and the activities of her organization for patients and their families ‘Everything for Her,’ I would see her real power – to move things forward and away from desperation and defeat. Vesna used her academic expertise in economy to run a charity organization in a most successful way – ‘Everything for Her’ organized several nation-wide actions that built up awareness about the need for social and psychological support to patients with cancer, and established the facilities and infrastructure for providing such help where the official health care system had failed. We collaborated on a joint project to create the Croatian register of clinical trials, so that patients and their families would be able to find out about new therapies and contact local physicians about possible inclusion in trials. She was also a firm supporter of evidence-based medicine, and asked us and the colleagues from the Croatian branch of the Cochrane Organization to provide the most recent guidelines on support therapies for patients with cancer. She used our one-line pieces of advice to supplement the exhibition of art photographs of women with cancer – she paid attention to the tiniest detail and would not stop until she was sure that the scientific language was converted into language understandable to all patients.

You have followed Vesna’s column over the last year and a half – nine stories told in her patient and persistent voice. In the last text, she could not continue her own story but put together a brief history of ‘Everything for Her’ and its achievements. She had her husband call me two days before her death to make sure that we got the text and approved of it. I am sure that she would also approve of our decision to continue publishing the stories of life and bravery in the face of disease. Vesna will not be with us but we asked Nika Matovac, Vesna’s daughter and our college – medical student at the Zagreb School of Medicine, to be the editor of a new series of ‘Patient Voice’ essays. The *Croatian Medical Journal* has always had a student editor and Nika will be the next one, tasked with collecting stories of patients and their families. So, we bid farewell until we meet Vesna again, and we sit down together to listen to new voices of life.

